# Complete chloroplast genome molecular structure, comparative and phylogenetic analyses of *Sphaeropteris lepifera* of Cyatheaceae family: a tree fern from China

**DOI:** 10.1038/s41598-023-28432-3

**Published:** 2023-01-24

**Authors:** Qingdi Hu, Renjuan Qian, Yanjun Zhang, Xiaohua Ma, Youju Ye, Xule Zhang, Lin Lin, Hongjian Liu, Jian Zheng

**Affiliations:** 1 Wenzhou Key Laboratory of Resource Plant Innovation and Utilization, Zhejiang Institute of Subtropical Crops, Wenzhou, 325005 Zhejiang China; 2 China National Bamboo Research Center, Hangzhou, 310012 Zhejiang China

**Keywords:** Ecology, Evolution, Plant sciences

## Abstract

*Sphaeropteris lepifera* is a tree fern in the Cyatheaceae, a family that has played an important role in the evolution of plant systems. This study aimed to analyze the complete chloroplast genome of *S. lepifera* and compared it with previously published chloroplast genomes Cyatheaceae family. The chloroplast genome of *S. lepifera* comprised 162,114 bp, consisting of a large single copy (LSC) region of 86,327 bp, a small single copy (SSC) region of 27,731 bp and a pair of inverted repeats (IRa and IRb) of 24,028 bp each. The chloroplast genome encoded 129 genes, comprising 32 transfer RNAs, 8 ribosomal RNAs, and 89 protein-coding genes. Comparison of the genomes of 7 Cyatheaceae plants showed that the chloroplast genome of *S. lepifera* was missing the gene *trnV*-UAC. Expansion of the SSC region led to the difference in the chloroplast genome size of *S. lepifera*. Eight genes, *atpI*, *ccsA*, *petA*, *psaB*, *rpl16*, *rpoA*, *rpoC1*, and *ycf2* have high nucleic acid diversity and can be regarded as potential molecular markers. The genes *trnG-trnR* and *atpB* were suitable for DNA barcodes between different communities of *S. lepifera*. The *S. lepifera* groups in Zhejiang Province probably diffused from Pingtan and Ningde, Fujian. The results will provide a basis for species identification, biological studies, and endangerment mechanism of *S. lepifera*.

## Introduction

Global climate change and human activities are threatening biodiversity, and the disappearance of species through extinction is a major ecological crisis^1,2^. There are perhaps 1–6 billion species on Earth, and the current extinction rate is 1000–10,000 times higher than the background extinction rate of 10^7^ species–years in the fossil record^3,4^. The International Union for Conservation of Nature (IUCN) now lists more than 35,000 (28%) surveyed plant and animal species as threatened with extinction. Some of these endangered plants are relict species that retain ancient and endemic genes that are important for maintaining genetic diversity due to their age and systematic isolation^5,6^. China is abundant in biodiversity, with endangered plant resources and many tertiary relict plants. However, due to global warming and human activities, the survival of some ancient and rare plants has been threatened, and some species have gone extinct^7,8^.

*Sphaeropteris lepifera*, originally named *Cyathea lepifera*, the leaves are three-pinnatifid; the petioles and blade are verrucous, and the pinna is usually 20–40 cm. The plants are primarily distributed in the Philippines, Japan, and China, and they mostly grow in patches at the edges of forests, on roadsides, or on sunny hillsides^9,10^. *S. lepifera* has medicinal and horticultural value, and is also of great significance in speciation, paleontology, and paleoclimate research^11^. However, because *S. lepifera* reproduces by spores and due to strong moisture dependence, difficulty in natural regeneration, and destructive deforestation, the wild *S. lepifera* distribution area and population size have decreased, and the species faces a greater risk of extinction than seed plants. Currently, *S. lepifera* has been included in the lists of China's second-class key protected wild plants and China's Rare and Endangered plants.

Much of the existing research on *S. lepifera* focuses on population structure, the ecological environment, reproduction, cultivation, and physiology. The natural growth of *S. lepifera* is sensitive to changes in light intensity; a high light environment prevents the regeneration of *S. lepifera* seedlings, and low light conditions lead to resource competition. The capture and utilization efficiency of *S. lepifera* is higher than those of *Alsophila spinulosa* and *A. podophylla*^12^. Niche models indicate that extremely low temperatures, long-term and short-term temperature stability, and precipitation seasonality are important abiotic environmental factors affecting the distribution of *S. lepifera*^13^. After the leaves of *S. lepifera* fall, toxic substances such as *p*-coumaric acid and (-)-3-hydroxy-*β*-ionone can be decomposed and released to inhibit the growth of other woody plants in tropical forests^14^. Huang et al. studied the gametophyte development and young sporophyte morphology of *S. lepifera*, in which the sperm-egg combination is the key link in sexual reproduction, and the development time of male and female gametes also strongly affects the survival and distribution of *S. lepifera*^15^. Wild *S. lepifera* populations in Taiwan area have suffered from epidemic plant diseases, and *Ophiodiaporthe cyatheae* is the pathogen that causes the wilting of *S. lepifera*^16,17^. To further protect *S. lepifera* resources, several in vitro culture systems have been established, with the goal to restore wild groups by artificial means^18,19^.

There are many reasons for plant endangerment, including reproductive difficulties, ecological destruction, reduction of the field community, and reduced resistance to disease^20^. Research on population genetic structure and diversity is an important approach to further explore the characteristics of endangered species^21,22^. The chloroplast genome has the advantages of simple structure, low molecular weight, multiple copies, and a moderate rate of evolution. It provides important molecular evidence for phylogenetic analysis, chloroplast genetic engineering, and molecular marker development^23,24^. The first chloroplast genome was sequenced in the 1980s, and over the past 30 years, approximately 100 chloroplast genomes of vegetable, fruit, grain, oil, and starch/sugar crops have been sequenced^25^. Plant chloroplast genomes generally exhibit conserved gene contents and sequences, but some plants (e.g., Leguminosae, Compositae, and Lagerstroemia) show varying degrees of genomic upheaval such as loss of genes, introns, or inverted repeat (IR) regions, gene duplication, and large-scale rearrangements^26,27^. In a study of Monsteroideae, the synonymous substitution rate of 76 protein-coding genes was higher than the non-synonymous substitution rate, and seven protein-coding genes (*psbK*, *ndhK*, *ndhD*, *rbcL*, *accD*, *rps8,* and *ycf2*) were developed as molecular markers^28^.

Organelle genome study is a part of the whole genome project, and the genome information of *A. spinulosa* has been announced as a milestone, but genome information of *S. lepifera* is still lacking. The chloroplast genome of *S. lepifera* is an important basis for analyzing all genetic information and resource conservation^29^. We compared the chloroplast genome of *S. lepifera* with those of other six species of tree ferns, explored potential DNA molecular markers, and analyzed the genetic diversity of *S. lepifera* in 32 different geographical regions. The results will provide a reference for the study of phylogenetic relationships, species identification, and endangerment factors of the trees in the future.

## Results

### Structural characteristics of the chloroplast genome

**Figure 1:**
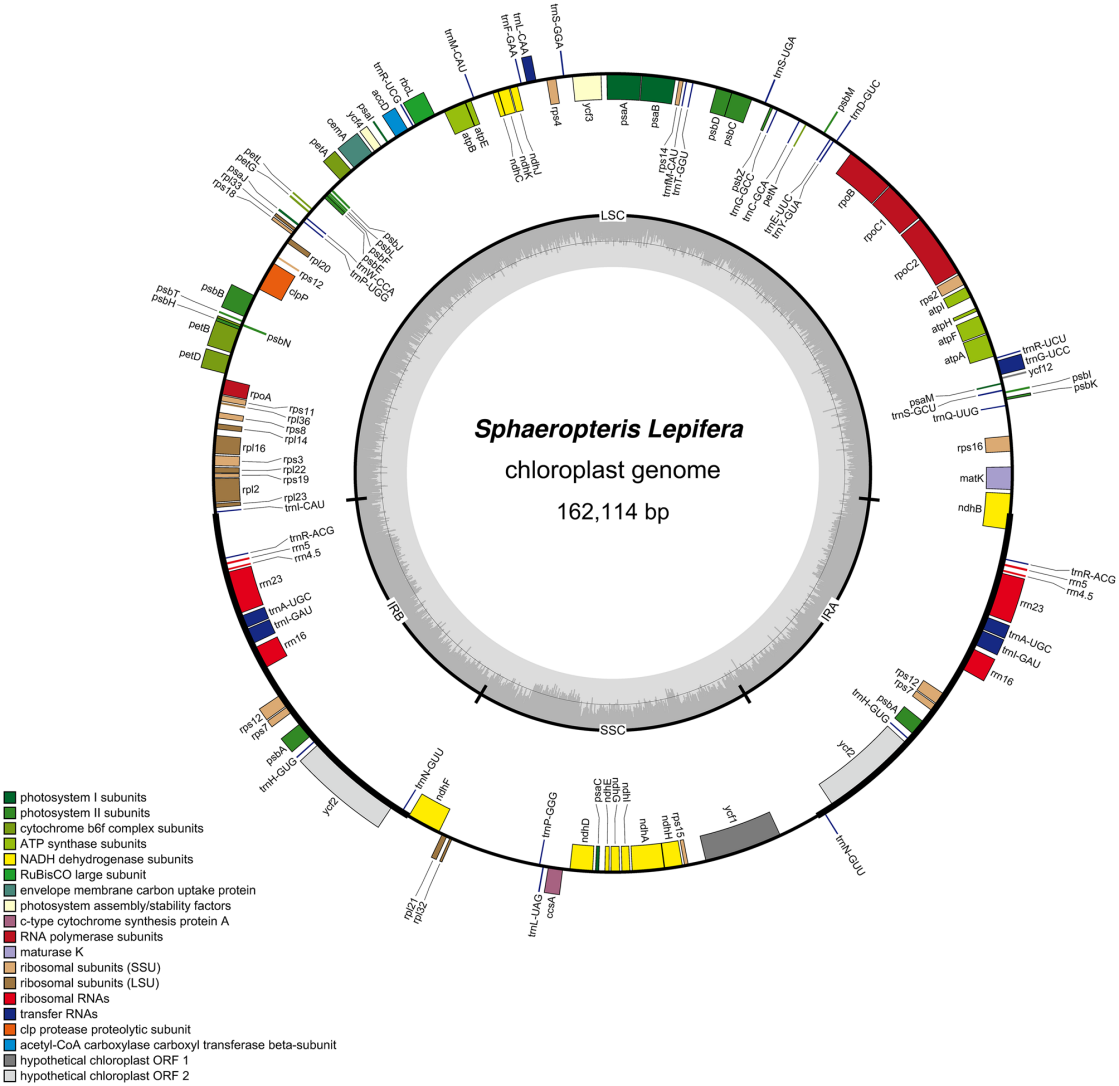
Chloroplast genome map of *S. lepifera*. Forward-coding genes are located outside the circle, and reverse-coding genes are located inside the circle. The gray circle inside represents the GC content.

After the total DNA extracted from *S. lepifera* was qualified, a valid library was constructed, and 86,794,248 paired ends were sequenced on an Illumina platform. The chloroplast genome of *S. lepifera* comprised 162,114 bp, and the NCBI accession number was NC_063825.1. The genome formed a covalently closed double-chain circular molecule with a typical four-segment ring structure (Fig. 1).

The chloroplast genome of *S. lepifera* included a pair of IR regions (24,028 bp), one large single copy (LSC) area (86,327 bp), and one small single copy (SSC) region (27,731 bp). The GC content in the whole chloroplast genome was 40.79%, with the highest GC content in the IR region (43.15%), and the contents in the LSC and SSC regions were 39.23% and 41.52%, respectively (Table 1).

**Table 1:** Chloroplast genome composition of *S. lepifera*.

Region	Length (bp)	A%	T/U%	C%	G%	GC%
Genome	162,114	29.36	29.86	20.92	19.87	40.79
LSC	86,363	29.73	31.01	20.30	18.94	39.23
SSC	27,731	29.73	28.75	21.72	19.80	41.52
IRa	24,028	28.87	27.98	21.03	22.12	43.15
IRb	24,028	27.98	28.87	22.12	21.03	43.15

### Gene annotation of the chloroplast genome

A total of 129 genes, comprising 32 tRNAs, 8 rRNAs, and 89 mRNAs, were annotated in the chloroplast genome of *S. lepifera*. Among the 116 non-repetitive genes, there were 4 rRNA genes, 27 tRNA genes, and 85 protein-coding genes. There were 48 photosynthesis-related genes, 57 self-replicating genes, six other functional genes, and five open reading frames. Among all the genes, 12 had one intron (10.34%), three had two introns (2.59%), and 11 had two copies (9.48%) (Table 2).

**Table 2:** Gene classification of *S. lepifera*.

Category	Group of genes	Gene name
Photosynthesis	Photosystem I	*psaA*,*psaB*,*psaC*,*psaI*,*psaJ*,*psaM*
	Photosystem II	*psbA*(2),*psbB*,*psbC*,*psbD*,*psbE*,*psbF*,*psbH*,*psbI*,*psbJ*,*psbK*,*psbL*,*psbM*,*psbN*,*psbT*,*psbZ*
	NADH dehydrogenase	ndhA*,ndhB*,*ndhC*,*ndhD*,*ndhE*,*ndhF*,*ndhG*,*ndhH*,*ndhI*,*ndhJ*,*ndhK*
	Cytochrome b/f complex	*petA*,petB*,petD*,*petG*,*petL*,*petN*
	ATP synthase	*atpA*,*atpB*,*atpE*,atpF*,*atpH*,*atpI*
	Rubisco	*rbcL*
	Photochlorophyllide reductase	*chlB*,*chlL*,*chlN*
Self-replication	Large ribosomal subunit	*rpl14*,rpl16*,rpl2*,*rpl20*,*rpl21*,*rpl22*,*rpl23*,*rpl32*,*rpl33*,*rpl36*
	Small ribosomal subunit	*rps11*,rps12**(2),*rps14*,*rps15*,rps16*,*rps18*,*rps19*,*rps2*,*rps3*,*rps4*,*rps7*(2),*rps8*
	RNA polymerase	*rpoA*,*rpoB*,rpoC1*,*rpoC2*
	rRNAs	*rrn16*(2),*rrn23*(2),*rrn4*.5(2),*rrn5*(2)
	tRNAs	*trnA*-UGC*(2),*trnC*-*GCA*,*trnD*-*GUC*,*trnE*-*UUC*,*trnF*-*GAA*,*trnG*-*GCC*,*trnG*-UCC*,*trnH*-*GUG*(2),*trnI*-*CAU*,*trnI*-GAU*(2),*trnL*-CAA*,*trnL*-*UAG*,*trnM*-*CAU*,*trnN*-*GUU*(2),*trnP*-*GGG*,*trnP*-*UGG*,*trnQ*-*UUG*,*trnR*-*ACG*(2),*trnR*-*UCG*,*trnR*-*UCU*,*trnS*-*GCU*,*trnS*-*GGA*,*trnS*-*UGA*,*trnT*-*GGU*,*trnW*-*CCA*,*trnY*-*GUA*,*trnfM*-*CAU*
Other genes	Maturase	*matK*
	Protease	clpP**
	Envelope membrane protein	*cemA*
	Acetyl-coa carboxylase	*accD*
	c-type cytochrome synthesis gene	*ccsA*
	Translation initiation factor	*infA*
	Conserved open reading frames	*ycf1*,*ycf12*,*ycf2*(2),ycf3**,*ycf4*

Gene*: Gene with one intron; Gene**: Gene with two introns; and Gene (2): Number of copies of multi-copy genes.

### Chloroplast genome codon analysis

**Figure 2:**
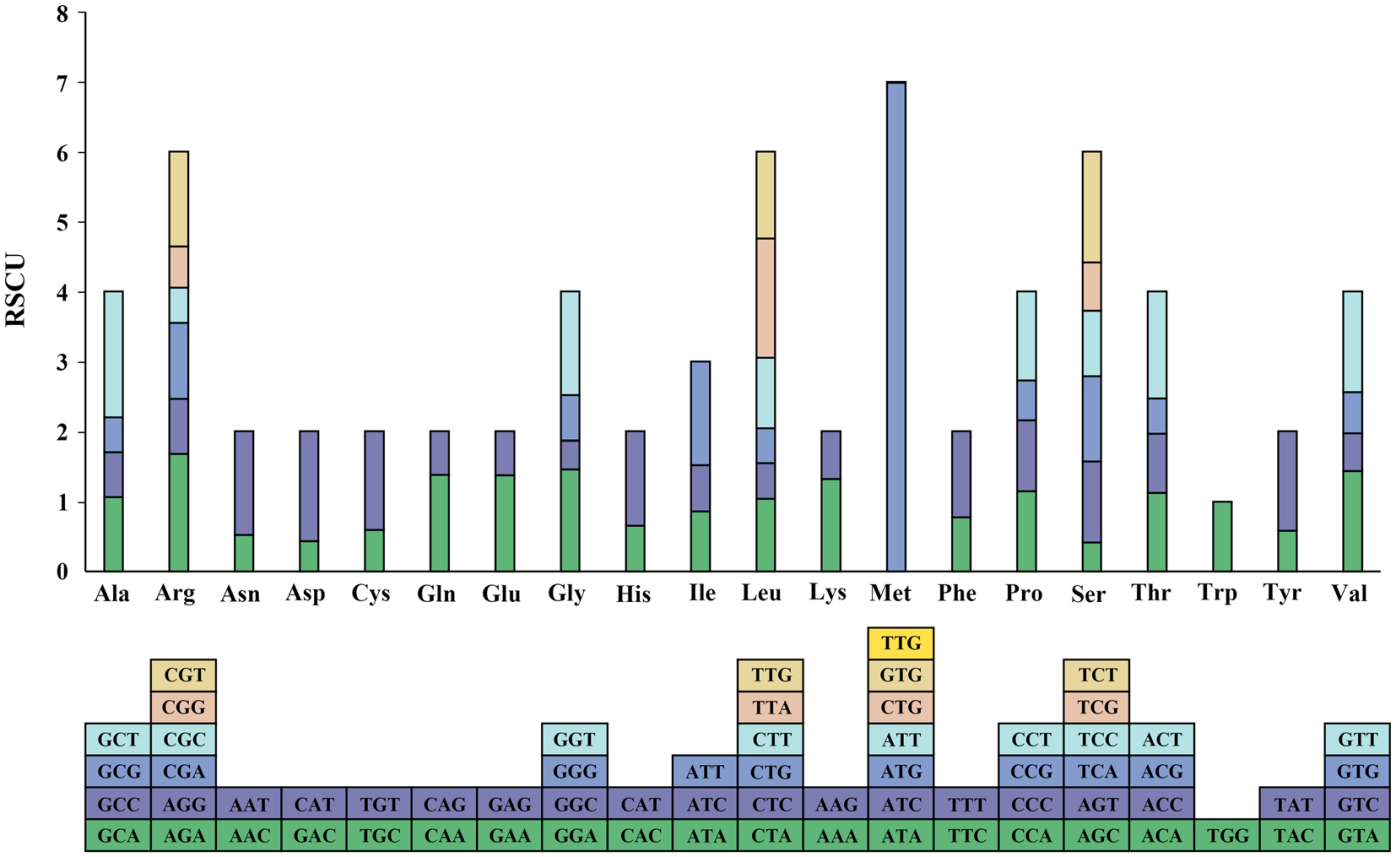
Relative synonymous codon usage of *S. lepifera*. The bottom square represents all codons encoding each amino acid, and the height of the upper column represents the sum of RSCU values of all codons.

We performed codon preference analysis on 85 protein-coding genes and found that AUU appeared the most frequently, 945 times (3.93%), while GUG appeared the least frequently, once (0.0042%). The number of Leucine (Leu) codons was 2472, accounting for 10.29% of the total. Codons encoding Cysteine (Cys) were the least numerous (269), accounting for 1.12% of the total (see Supplementary Table S1 online). Among the 65 codons, 34 codons (52.31%) had RSCU values greater than 1. In addition, 8048 codons (33.49%) ended in C or G, and 15,985 codons (66.51%) ended in A or U (Fig. 2).

### Repeat sequence analysis of the chloroplast genome

**Figure 3:**
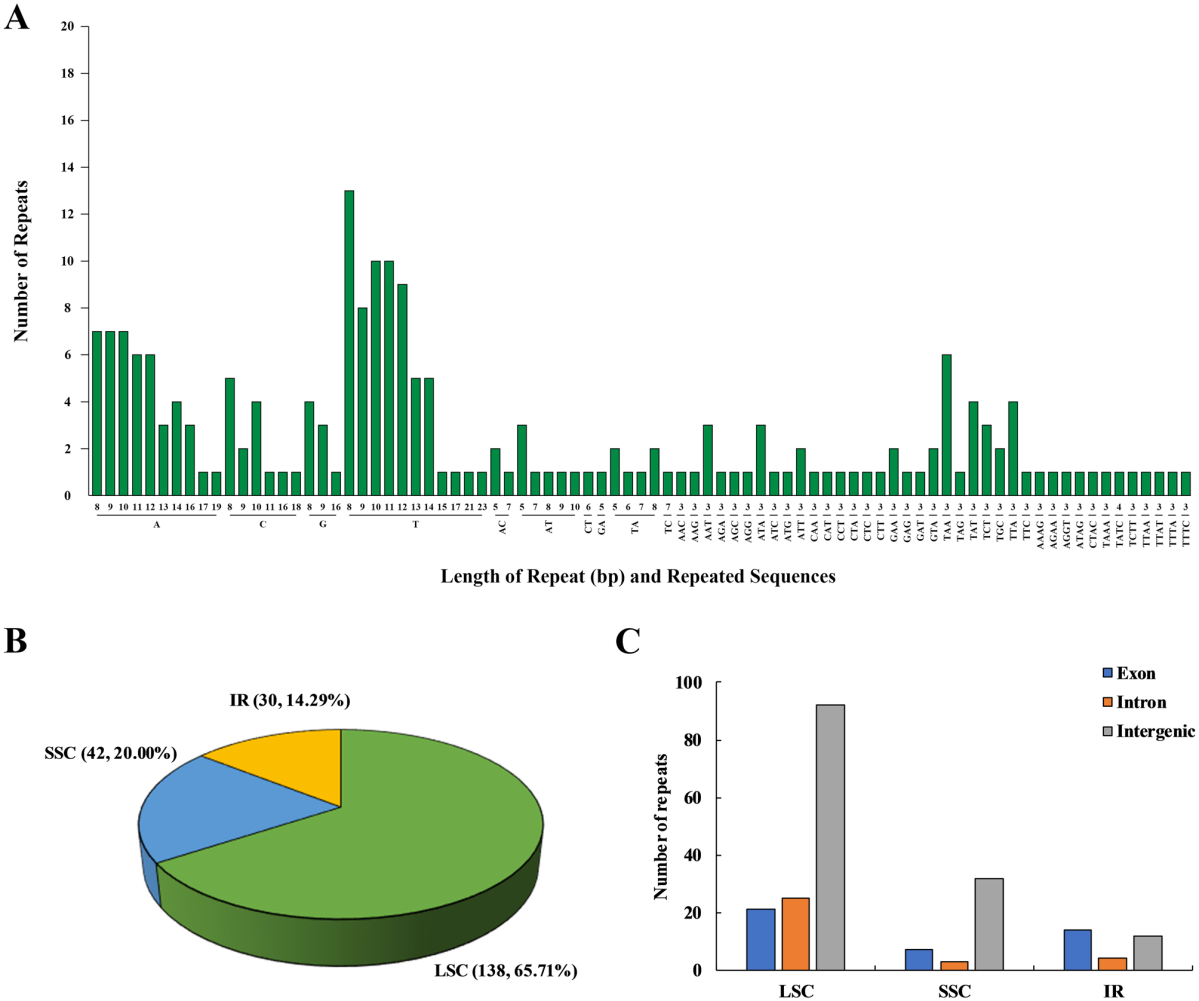
SSR analysis of *S. lepifera*. (**A**) SSR type. (**B**) The number of SSR in different regions. (**C**) Distribution region of SSR in genes.

A total of 210 single sequence repeats (SSRs) were identified in the chloroplast genome, comprising 131 mononucleotide repeats, 19 dinucleotide repeats, 48 trinucleotide repeats, and 12 tetranucleotide repeats, and 113 SSRs were larger than 10 bp. The longest SSR was a single base T repeat, with a length of 23 bp. There were 148 SSRs based on A/T, accounting for 86.96% of all SSRs (Fig. 3A). The IR region contained 30 SSRs (14.30%); the LSC region contained 138 (65.70%), and the SSC region contained 42 (20.00%) (Fig. 3B). Further analysis showed that 42 SSRs were in exons (20.00%); 32 SSRs were in intron regions (15.24%); and 136 SSRs were in intergenic regions (64.76%) (Fig. 3C). In addition to SSRs, there were 120 interspersed repeats in the chloroplast genome of *S. lepifera*, among which the 30 bp sequence was the most, 35 (28.93%). There was only one repetition at 48 bp and 58 bp that were palindromic and forward, respectively. Among all interspersed repeats, there were 49 forward repeats (40.50%), 55 palindromic repeats (45.45%), 11 reverse repeats (9.09%), and six complementary sequences (4.96%) (see Supplementary Fig. S1 online).

### Comparative analysis of chloroplast genomes

**Figure 4:**
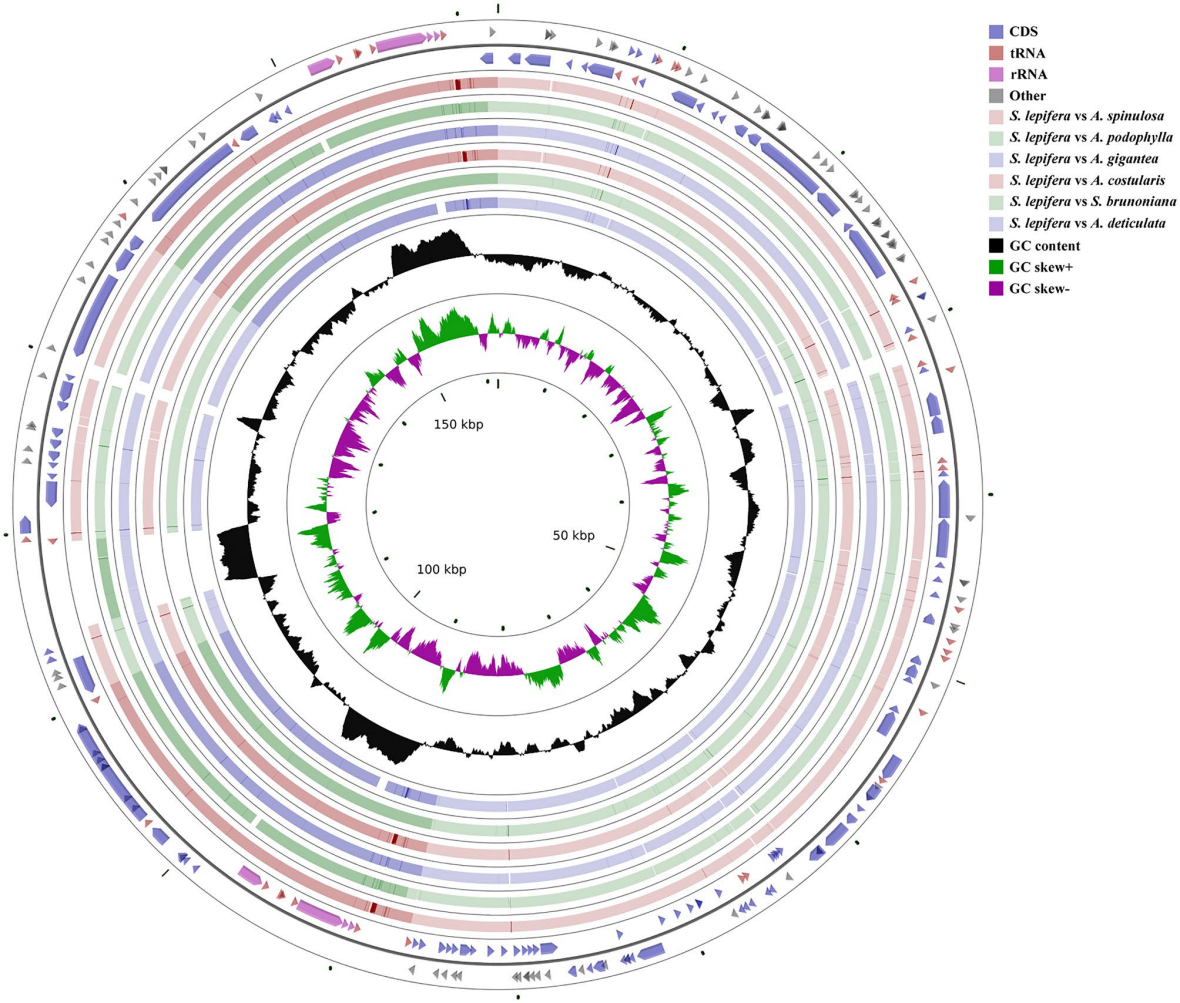
Graphic view of the chloroplast genome structures. The outermost circle shows the gene length, and the second outer circle indicates gene direction. The inner circles represent results of similarity with other reference genome alignments. The black circle represents GC content; green represents GC-skew+; and purple represents GC-skew−.

The conserved and highly mutated regions of the chloroplast genome can be visualized through the chloroplast genome structure with relatively close sequence consistency. The chloroplast genomes of *S. lepifera* (NC_063825.1), *Sphaeropteris brunoniana* (NC_051561.1), *Alsophila spinulosa* (NC_012818.1), *Alsophila podophylla* (NC_038150.1), *Alsophila gigantea* (NC_044079.1), *Alsophila costularis* (NC_044080.1), and *Alsophila denticulate* (NC_058591.1) were compared and analyzed. The consistency between *S. lepifera*, *S. brunoniana*, *A. gigantea*, and *A. podophylla* was higher than in other *Alsophila* species (Fig. 4). The chloroplast genomes of seven species of tree ferns ranged from 154,046 bp to 166,151 bp. The longest LSC region was in *A. gigantea* (92,315 bp); the longest SSC region was in *S. lepifera* (27,731 bp); the longest IR region was in *A. podophylla* (28,874 bp); and the number of genes was 129–133 (see Supplementary Table S2 online).

### Contraction and expansion of the IR region

**Figure 5:**
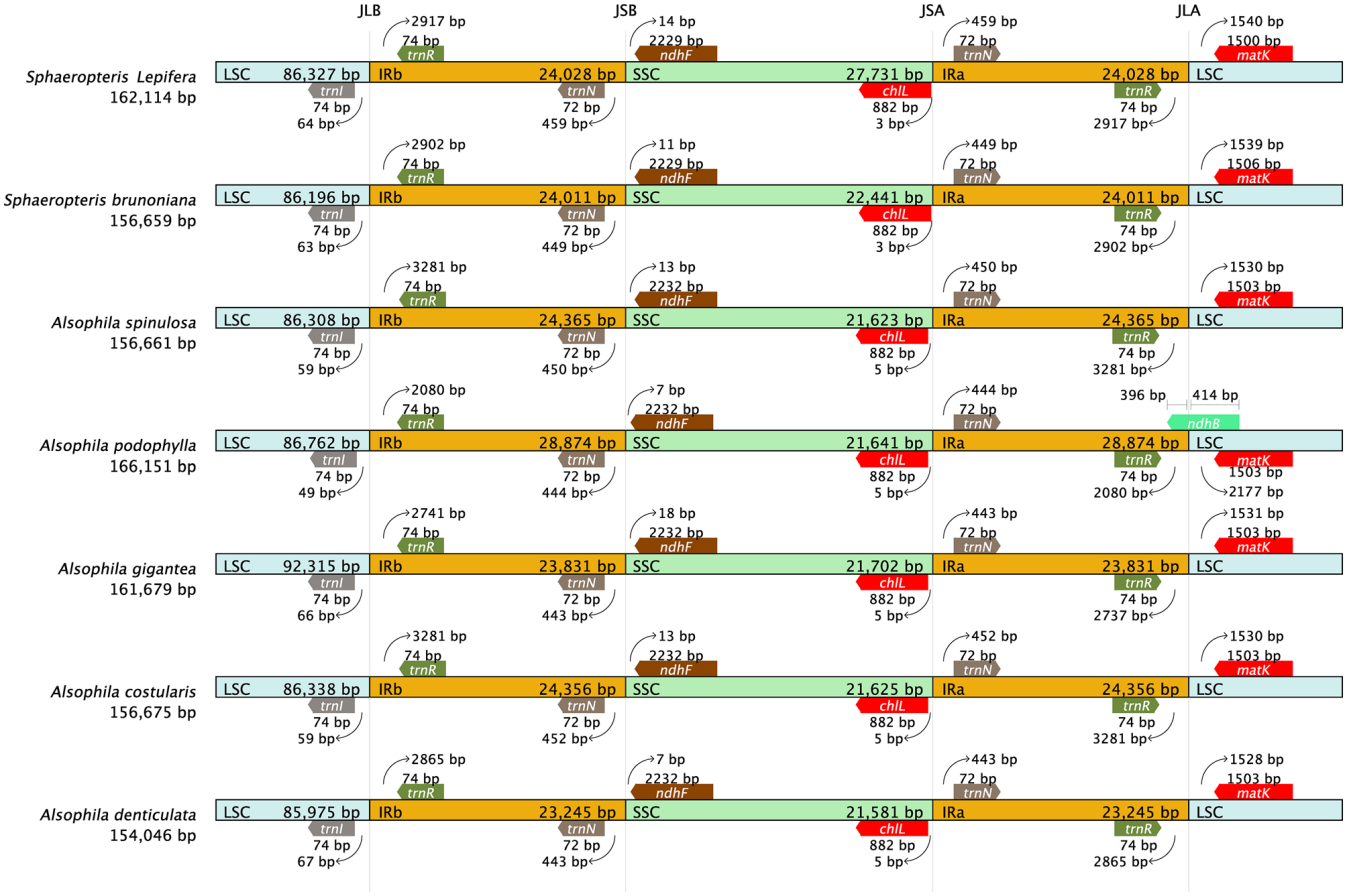
IR and SC boundary analysis.

The contraction and expansion of the IR boundary have led to changes in the copy number of related genes or the generation of pseudogenes in the boundary region; this is also the main reason for the variation of chloroplast genome size. The chloroplast genomes of seven tree fern species had the same gene arrangement at the IR/SC boundary, *trnl*, *trnR*, *trnN*, *chlL*, and *ndhF* were relatively conserved. The four genes, *trnl*, *trnR*, *trnN*, and *chlL*, had the same length. In *S. lepifera*, *ndhF* had the same length as in *S. brunoniana* (2229 bp), and the length of *matK* was the shortest (1500 bp) (Fig. 5). Mauve analysis of the chloroplast genomes of seven tree ferns detected no large fragment gene rearrangements, indicating a collinear relationship. Interestingly, the *trnV*-UAC gene was missing from *S. lepifera* (see Supplementary Fig. S3 online).

### Genome sequence divergence among tree fern species

**Figure 6:**
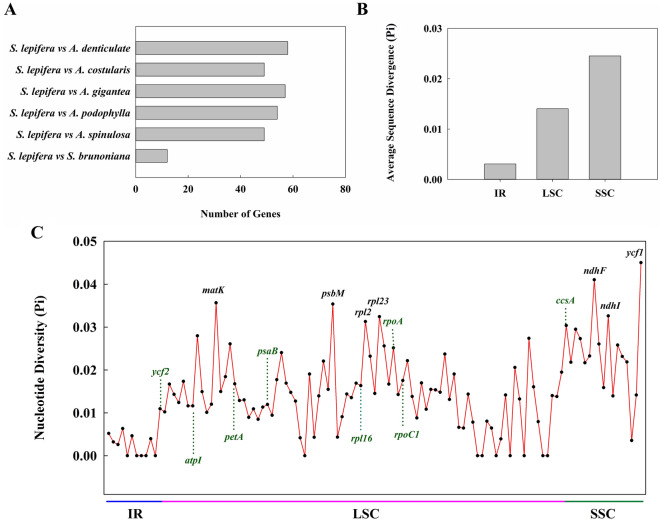
Nucleic acid diversity analysis. (**A**) Number of purified selective gene mutations between *S. lepifera* and other tree ferns. (**B**) Average nucleic acid diversity in different regions. (**C**) Nucleotide diversity analysis of genes.

*Ka/Ks* analysis showed that 58 genes in *S. lepifera* vs. *A. denticulate*, 57 genes vs. *A. gigantea*, 54 genes vs. *A. podophylla*, 49 genes vs. A. spinulosa, 49 genes vs. *A. costularis*, and 12 genes vs. *S. brunoniana* possessed base mutations by purification selection (*P* < 0.05) (Fig. 6A; Supplementary Table S3). Among all the mutated genes, *rpoB*, *matK*, *psbD*, *rbcL*, *petA*, *chlB*, *rps12*, *chlL*, and *ndhB* all had purified and selected mutations in the seven tree ferns. Gene *rpl16* was mutated only between *S. lepifera* and *A. podophylla*. *ndhc* and *petL* were mutated only between *S. lepifera* and *A. gigantea*, and *rpl22* was mutated between *S. lepifera* and *A. denticulate* (see Supplementary Table S3 online).

Nucleic acid diversity (Pi) can reveal the variation of nucleic acid sequences of different species, and regions with a high degree of variation can provide potential molecular markers for population genetics. We compared the nucleic acid diversity in IR, LSC, and SSC regions of seven tree fern chloroplast genomes, and the average Pi in LSC was 0.014. The largest difference was in the SSC region (0.025), and the smallest difference was in IR (0.003) (Fig. 6B). The mean Pi of nucleotide diversity in *S. lepifera* was 0.014, and 52 genes had larger values than the average, comprising 38 genes in the LSC and 14 genes in the SSC. In addition, Pi values of *ycf1*, *ndhF*, *matK*, *psbM*, *ndhI*, *rpl23*, *rpl2*, and *ccsA* were greater than 0.03; 80 genes were greater than 0.01; and 102 genes were greater than 0 (Fig. 6C).

### Genetic evolutionary analysis

**Figure 7:**
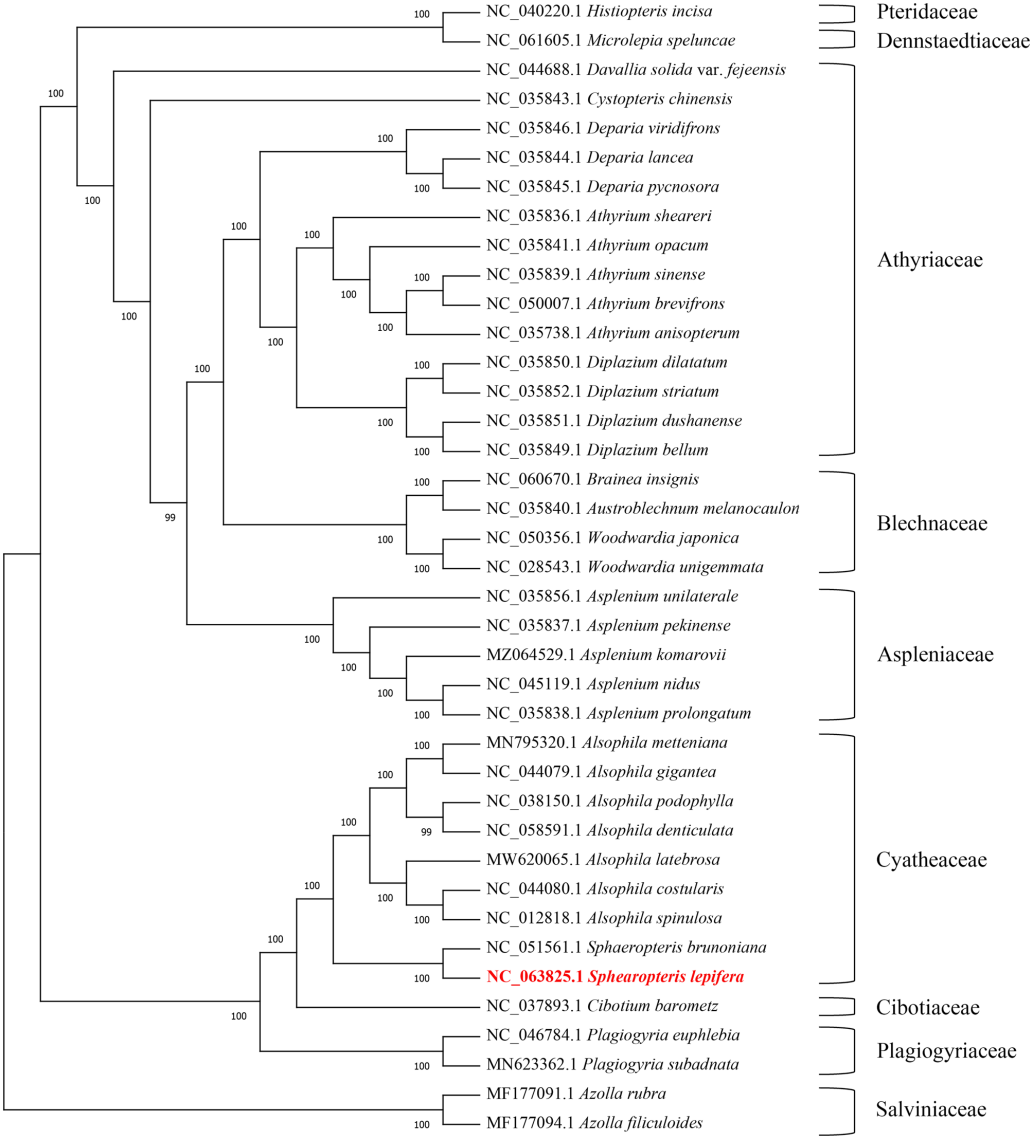
Phylogenetic tree based on the whole chloroplast genome. Phylogenetic tree constructed from 39 species complete chloroplast genome sequences using maximum likeliood (ML) method. Bootstrap values are marked above the branches.

To clarify the phylogenetic and evolutionary relationships of *S. lepifera*, 39 plants including *S. lepifera* were selected to construct a phylogenetic tree. The results showed that *S. lepifera* was closely related to *S. brunoniana*, *A. spinulosa*, *A. costularis*, *A. latebrosa*, *A. denticulate*, *A. podophylla*, *A. gigantea*, and *A. metteniana* (Fig. 7).

**Figure 8:**
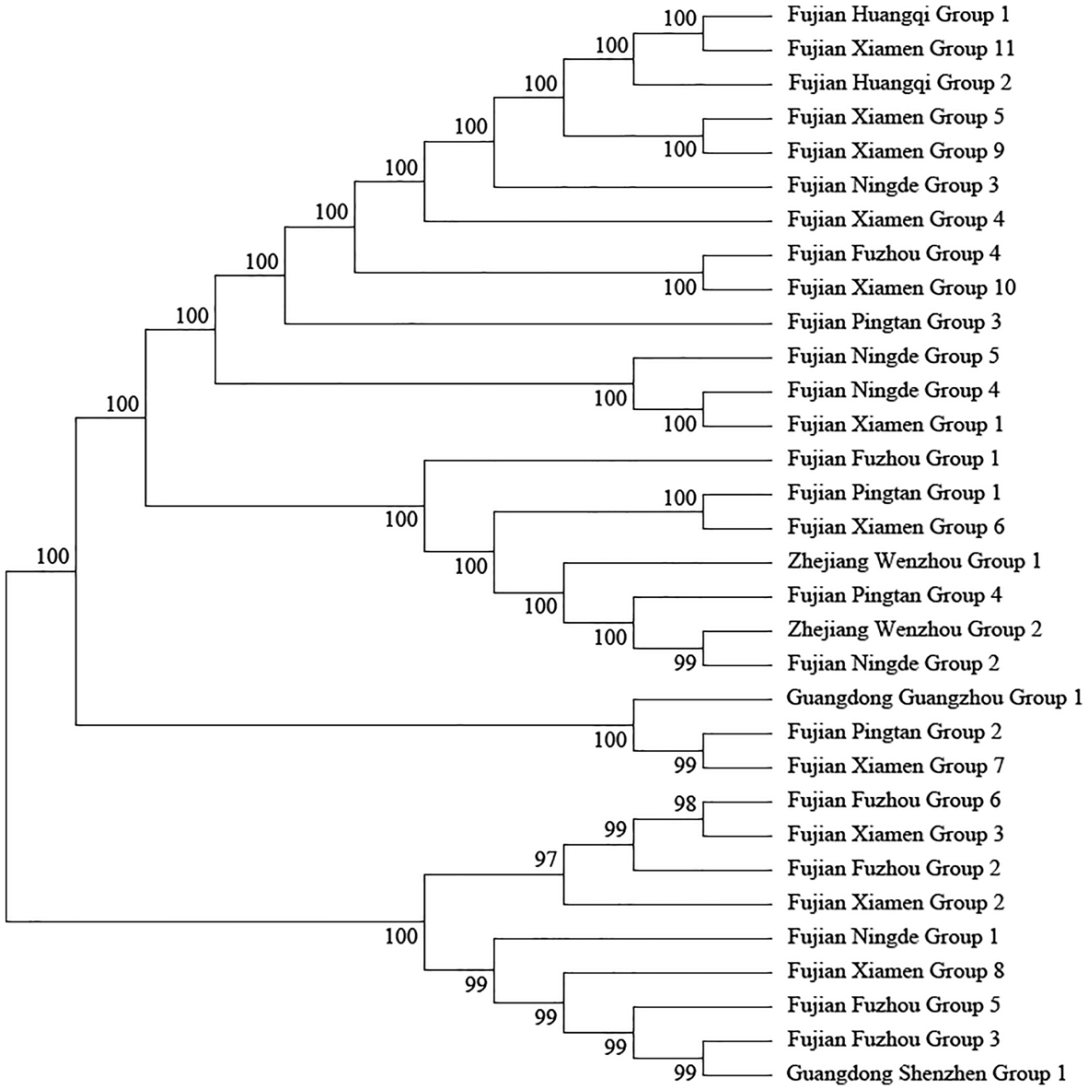
Phylogenetic tree of *S. lepifera* groups.

We collected 32 *S. lepifera* groups from different geographic regions and selected nine genes or gene intervals, including *matK*, *rbcL*, *rps4*, and *trnG-trnR*, for testing neutral molecular evolution. Tajima's D* values, Fu and Li's D* values, and Fu and Li's F* values of these genes were all negative, and the P values were not significant, in line with the neutral evolution mode (see Supplementary Table S4 online). The sequences were merged into joint fragments to construct an evolutionary tree, indicating that the two *S. lepifera* groups in Zhejiang Province were most closely related to *S. lepifera* groups in Ningde and Pingtan, Fujian Province (Fig. 8).

## Discussion

Studies have shown that chloroplast genome size in plants ranges from 15,553 bp in *Asarum minus* to 521,168 bp in *Floydiella terrestris*. The number of the genes encoded by the chloroplast DNA in different plant species ranges from 0 to 315, and the structure is very conservative (NCBI)^30,31^. The chloroplast genome of *Nicotiana tabacum* was the first chloroplast genome to be sequenced in higher plants^32^. In this study, the chloroplast genome of *S. lepifera* after sequencing, assembly, and correction was 162,114 bp, presenting a typical four-section structure (Fig. 1). The GC content was 40.79%, similar to the GC content reported in other tree ferns^33,34^. GC pairs with three hydrogen bonds were more stable than AT base pairs containing two hydrogen bonds, which results in genes with low GC contents being more easily transcribed than those with the converse^35^. Among angiosperms, the chloroplast genome GC content averaged 37.5% in the genus *Peucedanum*, 35.59% in the genus *Paphiopedilum*, and 38.33% in the genus *Agropyron*^36–38^. The relatively high chloroplast genome GC content of 40.79% in *S. lepifera* may present a greater risk of environmental adaptation compared to other species.

A total of 129 genes were encoded in the chloroplast genome of *S. lepifera*. In addition, pseudogenes *ycf66* and *trnT*-UGU were also detected and were consistent with *S. brunoniana*, *A. spinulosa*, *A. gigantea*, and *A. denticulate*. There was no *ycf66* or *trnT*-UGU, and only the *ndhB* pseudogene existed in *A. podophylla*. Most of the photosynthesis-related genes in mature chloroplasts are transcribed by the bacterial-type multi-subunit RNA polymerase (PEP) and the T3/T7 phage-type RNA polymerases (NEP), and transcription initiation factors (sigma factors, SIGs) are essential for specific binding of PEP to promoters of the corresponding genes. The tRNA encoded by *trnV*-UAC is accumulated in a sig2-dependent manner, and *trnE* and *trnD* are sequentially arranged on the plastid genome and transcribed together in chloroplasts of tobacco^39,40^. The deletion of the *trnV*-UAC between the *ndhC* and *atpE* in the LSC region of *S. lepifera* may be the result of natural selection to adapt to the environment (Supplementary Fig. S3). In addition, because the IR region of *S. lepifera* contained all rRNAs and five tRNAs, the GC content of the IR was higher than in the LSC and SSC regions, similar to other higher plant species^41,42^.

Due to the degeneracy of codons, each amino acid corresponds to at least one codon and at most six codons. There are great differences in genomic codon utilization among different species and organisms, and this preference is the comprehensive result of natural selection, mutation, and genetic drift^43,44^. With the rapid development of chloroplast gene transformation, many studies have reported the applicability of chloroplast transgenic technology to plants, and the analysis of codon usage patterns can provide support for the construction of mature and stable chloroplast transgenic systems^45,46^. Among the 34 preference codons in *S. lepifera*, 30 had A or U as the third base, and only UUG, GGG, AUG, and UGG end with G (Fig. 2; Supplementary Table S1), reflecting the A/T preference of codons, similar to most chloroplast genomes of angiosperms^47,48^. The common presence of C to U RNA editing events in higher plant chloroplasts, coupled with adaptation due to natural selection by environmental factors, which may be responsible for biased codon production in penstemon.

As an important molecular marker, SSR variation in chloroplasts has a larger classification distance than nuclear or mitochondrial microsatellites and has been widely used in plant population genetics, in studies of polymorphism, and in evolutionary studies^49,50^. The dominant SSR type of *S. lepifera* was mononucleotide repeats, with trinucleotide repeats accounting for 36.64% of mononucleotide repeats, and dinucleotide and tetranucleotide repeats were fewer. The SSR containing A/T repeats accounted for 86.96% of all SSR types (Fig. 3), consistent with the distribution characteristics previously reported in tree ferns^51^. This has also been reported in chloroplast genome studies of *Abelmoschus* L., *Callitropsis funebris*, and other plants^52,53^. Most of the SSRs were in the intergenic region (64.76%), which could be the result of genetic variation due to the higher rate of intergenic mutations than the region coding for the intergenic mutation rate. Among the coding genes, the gene with the most SSRs was *ycf2* with 8 SSRs, followed by *clpP*, *rpl2*, *trnL*-GAU, *ycf1*, and *ycf3* with 4 SSRs (Supplementary Fig. S2). Similar results have been reported in *Dysphania ambrosioides*, *Globba lancangensis*, and *Cremastra appendiculata*^54,55^. These high SSR density genomic regions can be exploited as potential molecular markers in phylogenetic studies.

The chloroplast genomes of *S. lepifera* and the other six tree ferns were collinear. In addition, no rearrangement has occurred. The sequence of genes was highly conserved, and the sequence variation of the non-coding region was generally higher than that of the coding region (Fig. 5). The boundary of the IR/SC region was conservative, with *trnL* in the LSC region, *trnN* and *trnR* in the IR region, and *ndhF* and *chlL* in the SSC region. However, the variation of gene stretching between IR/SC differed among species. *A. spinulosa trnR* was the farthest from the boundary at 3218 bp, while *A. podophylla matK* was the farthest to the boundary at 2177 bp. The distances of six genes of *S. lepifera* to the boundary were similar to those of *S. brunoniana*, indicating that there were certain species and group specificity of tree ferns at the IR/SC boundary. The SSC region of *S. lepifera* was the longest, with an increase of 6150–5290 bp compared with the other six tree ferns, resulting in the difference in chloroplast genome size. This may also be the reason for the high efficiency of *S. lepifera* in capturing and utilizing light resources. The distances of *ndhF* and *chlL* from the boundary were 14 bp and 3 bp, respectively, values that were not significantly different, indicating that the chloroplast genome expansion of *S. lepifera* was conservative in the gene coding region as in *Cypripedium tibeticum* and *Anubias heterophylla*^56,57^. The differences in the combined four boundary regions do not clearly and accurately reflect their phylogenetic processes, suggesting that the evolutionary development of *S. lepifera* may also depend on changes in other genes.

The low *Ka/Ks* ratio at the chloroplast genome level of *S. lepifera* indicated that most genes have undergone purifying selection to maintain conserved function (Supplementary Table S3). Environmental factors, such as solar radiation and temperature, can affect mutation rates, metabolism and growth rates^58^. Previous studies have shown that cold temperature extremes, temperature stability over long- and short-terms, and the seasonality of precipitation were among the most important abiotic environmental factors affecting the distribution of *S. lepifera*^13^. Photosynthetic organisms generally have a much larger number of genes, usually 30,000, and many of these genes act in the photosynthetic leaf tissue^59^. *psbB* and *psbD* in *S. lepifera*, which have relatively low *Ka/Ks* values compared to other tree ferns, are important components of photosynthetic system II (PSII). *psbD* encodes the reaction center protein D2 of PSII, and *psbB* encodes the PSII chlorophyll-binding protein of 47 kDa (CP47). It contributes with chlorophyll-binding protein 43 kDa (CP43) in the formation of the inner light-harvesting complex^60^, and therefore any lethal mutation may lead to impaired photosynthetic function of leaf cells. The genes with relatively high *Ka/Ks* were *cemA*, *ycf2* and *ycf3*, with *Ka/Ks* ranging from 0.5498 to 0.6241. Purifying selection can eliminate deleterious mutations in the population, and positive selection of genes is related to specific environments^61^. Genes with *Ka/Ks* greater than 1 were not observed in *S. lepifera* (*P* < 0.05), which may be related to the adaptive evolution and narrow distribution range of *S. lepifera*.

Phylogenetic analysis based on the whole chloroplast genome sequence showed that *S. lepifera* and *S. brunoniana* branches were closest, followed by *A. spinulosa* and *A. costularis*, consistent with phylogenetic analysis using single-copy nuclear gene sequences from transcriptomes. Only *ycf1* was greater than 0.001 in IR, indicating that highly variable genes in the chloroplast genome of *S. lepifera* were mostly located in the SSC and LSC. Genes such as *ycf1*, *ycf2*, *psbA*, *matK*, and *ndhF* have been detected as hypervariable regions in different plants^62,63^. We conducted molecular phylogenetic analysis for each gene with Pi greater than 0.01, and found that the branching reliability of *atpI*, *ccsA*, *petA*, *psaB*, *rpl16*, *rpoA*, *rpoC1*, and *ycf2* was more than 70% (Supplementary Fig. S4). Based on these results, eight genes with high sequence bias including *atpI*, *ccsA*, *petA*, psaB, *rpl16*, *rpoA*, *rpoC1*, and *ycf2* are good sources for interspecific phylogenetic analysis. We found 31 haplotypes in *trnG-trnR* by analyzing *S. lepifera* in different geographical populations, and the values of Hd and Pi were 0.9980 and 0.01539, respectively, with a variation rate of 8.36%. The *atpB* gene produced 10 haplotypes, with Hd and Pi being 0.706 and 0.00267, respectively, and the variation rate was 1.53% (Supplementary Table S4). The genes *rps4*, *matK*, *psbA*-*trnH*, *proB*-*psbZ*, *atpA*, *ndhF*, and *rbcL* were not suitable as intraspecies DNA barcodes for different *S. lepifera* groups.

Wild *S. lepifera* populations were first found in Cangnan, Taishun, and Longwan of Zhejiang in 2015, the northernmost distribution of *S. lepifera* in China. Currently, only one wild *S. lepifera* group remains in Zhejiang. We hope to expand the *S. lepifera* group through artificial spore reproduction and reintroduction to the wild. The group distribution of *S. lepifera* in Zhejiang was closely related to the *S. lepifera* groups in Ningde and Pingtan of Fujian. Therefore, we speculated that climate factors such as typhoons or human activities may have led to the spread of *S. lepifera* from Fujian to Zhejiang and promoted the group distribution boundary to move northward.

## Conclusions

Chloroplast DNA does not involve gene recombination in the process of transmission from parent to offspring, and thus, it has the characteristics of conservation and uniparental inheritance. The chloroplast genome of *S. lepifera* is of great significance for further study of chloroplast function, genetic diversity, population structure, evolutionary relationships, and molecular identification. In this study, we analyzed the *S. lepifera* chloroplast genome and compared it with the genomes of six other tree ferns. We found that the SSC and non-coding regions of *S. lepifera* were significantly different. The close evolutionary distance between *S. lepifera* and *S. brunoniana* was consistent with previous studies. The results also suggested that *S. lepifera* in Zhejiang might have diffused from Fujian *S. lepifera*. The findings provide eight genes including *atpI*, *ccsA*, *petA*, *psaB*, *rpl16*, *rpoA*, *rpoC1*, and *ycf2* as DNA barcode for future studies of genetics, biology, and endangerment factors of *S. lepifera* and other endangered plants.

## Methods

### Plant materials

The plant materials used in this study were obtained from the wild and permission was obtained to collect samples. The collection of plant materials also complied with institutional, national, or international guidelines. Fresh leaves of *S. lepifera* were collected from Cangnan, Wenzhou, Zhejiang Province, China (E 120° 60′, N 27° 39′) and stored at − 80 °C for chloroplast genome sequencing. It was identified by Professor Jian Zheng. Voucher specimens were deposited in Zhejiang Institute of Subtropical Crops, Wenzhou, Zhejiang Province, China (Voucher Code: W-2017-15). Fresh leaves from 32 wild *S. lepifera* groups were collected in Zhejiang, Fujian, and Guangdong; transported on dry ice; and stored at − 80 °C.

### Chloroplast genome sequencing and assembly

The total genomic deoxyribonucleic acid (DNA) was extracted from 100 mg of *S. lepifera* leaves using a Plant Genomic DNA Kit (Tiangen, Beijing, China)^64^. The DNA was fragmented by mechanical interruption (ultrasound), and a sequencing library was constructed. The qualified library was sequenced on an Illumina NovaSeq 6000 platform (Illumina NovaSeq 6000 platform, San Diego, CA, USA; Sequencing company: Genepioneer Co., Ltd., Nanjing, China), and the sequencing read length was 150 bp. The fastp v0.20.0 (https://github.com/OpenGene/fastp) software was used to filter the raw data to obtain clean data. Core module assembly adopted the SPAdes v3.10.1 (http://cab.spbu.ru/software/spades/) software to assemble the *S. lepifera* chloroplast genome^65^; kmer used 55, 87, and 121; and the assembly did not depend on the reference genome. Quality control analysis was performed on the assembled chloroplast genome to ensure the accuracy of the assembly results.

### Annotation of the chloroplast genome

Prodigal v2.6.3 was used to annotate the CDS of chloroplasts (https://www.github.com/hyattpd/Prodigal); hmmer v3.1b2 software was used to predict rRNA (http://www.hmmer.org/); and aragorn v1.2.38 was used to predict tRNA (http://130.235.244.92/ARAGORN/). Then gene sequences extracted from related species published in the NCBI database and the assembled sequences were compared with BLAST v2.6 to obtain the second annotation results (https://blast.ncbi.nlm.nih.gov/Blast.cgi). The genes with different annotation results were manually checked to remove incorrect and redundant annotations, to determine the boundaries of multiple exons, and to obtain the final annotation. Chloroplast genome mapping used OGDRAW (https://chlorobox.mpimp-golm.mpg.de/OGDraw.html).

### Chloroplast genome analysis

Software Codon W was used to analyze the codon usage of the genome (http://codonw.sourceforge.net/). Vmatch v2.3.0 software and Perl scripts were used to identify interspersed repeats (http://www.vmatch.de). MISA v1.0 software was used for SSR analysis (http://pgrc.ipk-gatersleben.de/misa/misa.html), with parameters 1–8 (single base repeated eight times or more), 2–5, 3–3, 4–3, 5–3, 6–3^66^. Mafft v7.310 software was used to compare gene sequences (https://mafft.cbrc.jp/alignment/software/), and KaKs_Calculator v2.0 software was used to calculate gene *Ka/Ks* values (https://sourceforge.net/projects/kakscalculator2/). DnaSP6.0 was used to calculate the Pi value of each gene (http://www.ub.edu/dnasp/). Analysis of chloroplast genome boundaries used the SVG module in Perl.

### Comparative analysis of chloroplast genomes

Comparative analysis of chloroplast genome structure of proximal species was performed using CGVIEW default parameters (http://stothard.afns.ualberta.ca/cgview_server/). The whole genome was used for evolutionary tree analysis; ring sequences were set at the same starting point; mafft v7.427 was used for multiple sequence comparison; and GTAGAMMA model with bootstrap analysis executed with 1000 replicates was used to construct the maximum likelihood evolutionary tree by RAxML V8.2.10 software (https://cme.h-its.org/exelixis/software.html). Genomic collinearity analysis was performed using Mauve software with default parameters (http://darlinglab.org/mauve).

### Population analysis

Total genomic DNA of *S. lepifera* was extracted using the Plant Genomic DNA kit (Tiangen Biotech, China). Chloroplast gene primers are shown in Table S5. The polymerase chain reaction (PCR) system was as follows (50 μL): genomic DNA (20 ng/µL), 1.0 µL; 10 × buffer (including 2.5 mM Mg^2+^), 5.0 µL; Taq polymerase (5 U/μL), 1.0 µL; dNTP (10 mM), 1.0 µL; F primer (10 μM), 1.5 µL; R primer (10 µM), 1.5 µL; and ddH_2_O, 39.0 µL. PCR parameters were as follows: 95 °C pre-denaturation for 5 min; 95 °C denaturation for 30 s, 58 °C annealing for 30 s, 72 °C extension for 1 min, and 72 °C final extension for 7 min, with 35 cycles. DNA products were recovered by electrophoresis and sequenced after purification.

MAGE7.0 software was used to compare the sequences of all materials, and the manual correction was carried out according to the sequencing map. Partial sequences with unreliable edges were removed, and the base ratio and the number of mutated bases were counted. Maximum likelihood (ML) was used to construct a molecular phylogenetic tree (Tamura-Nei model)^67^. The haplotype number (H), haplotype diversity (Hd), nucleotide diversity (Pi), and Tajima's D of the population were calculated using DnaSP6.0 software, and a neutrality test was performed.

### Ethics approval and consent to participate

All the plant materials in this study were obtained from the wild and permission was obtained to collect samples. This study protocol complies with relevant institutional, national, and international guidelines and legislation. This study protocol also complies with the IUCN Policy Statement on Research Involving Species at Risk of Extinction and the Convention on the Trade in Endangered Species of Wild Fauna and Flora.

## Supplementary Information

Supplementary Figures.

Supplementary Tables.

## Data Availability

The NCBI accession number of *S. lepifera* was NC_063825.1 (https://www.ncbi.nlm.nih.gov/nuccore/NC_063825.1). The raw genome sequence data have been deposited at the NCBI Sequence Read Archive with accession number PRJNA869881 (https://www.ncbi.nlm.nih.gov/bioproject/PRJNA869881). The associated Bio-Sample and SRA numbers were SAMN30330668 (https://www.ncbi.nlm.nih.gov/biosample/SAMN30330668/) and SRP392436 (https://www.ncbi.nlm.nih.gov/sra/SRP392436), respectively.

## Abbreviations

bp: Base pair
LSC: Large single copy
SSC: Small single copy
IR: Inverted repeat
IUCN: International Union for Conservation of Nature
A: Adenine
T: Thymine
G: Guanine
C: Cytosine
Leu: Leucine
Cys: Cysteine
RSCU: Relative synonymous codon usage
SSR: Single sequence repeat
PEP: Plastid‐encoded plastid RNA polymerase
NEP: Nucleus‐encoded plastid RNA polymerase
DNA: Deoxyribonucleic acid
PCR: Polymerase chain reaction
ML: Maximum likelihood
H: Haplotype number
Hd: Haplotype diversity
PSII: Photosynthetic system II
