# Supplementary material for: Complete chloroplast genome molecular structure, comparative and phylogenetic analyses of Sphaeropteris lepifera of Cyatheaceae family: a tree fern from China
Source: Sci Rep. 2023 Jan 24;13:1356. doi: 10.1038/s41598-023-28432-3 (PMC9873718; doi:10.1038/s41598-023-28432-3)
Supplement: Supplementary file 1 — Supplementary Figures. [file 41598_2023_28432_MOESM1_ESM.docx]

**
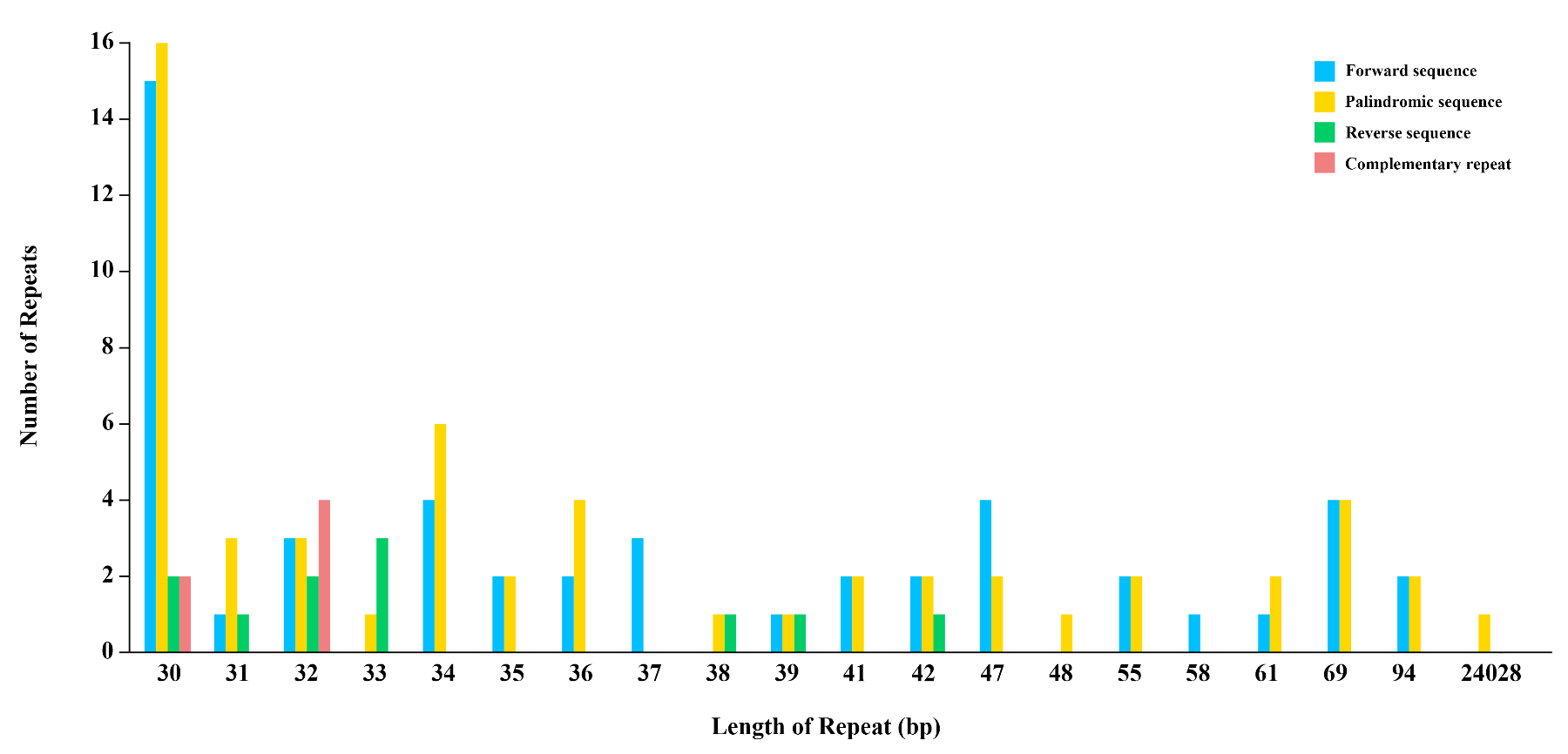
**

**Figure S1. Interspersed repeats analysis of *S. lepifera*.**





**Figure S2. The number of SSRs contained in chloroplast genes.**


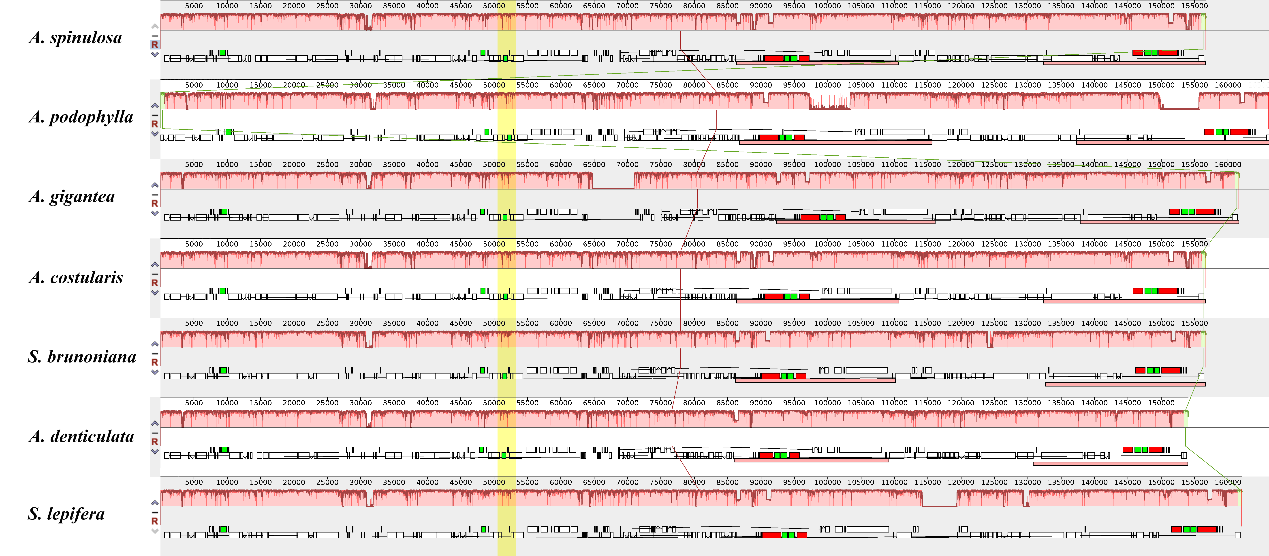


**Figure S3.** **Collinearity analysis of the chloroplast genome.** The long squares represent similarities between genomes. The lines between the long squares represent collinear relationships. The short squares represent the locations of genes in each genome. White represents CDS; green represents tRNA; and red represents rRNA.


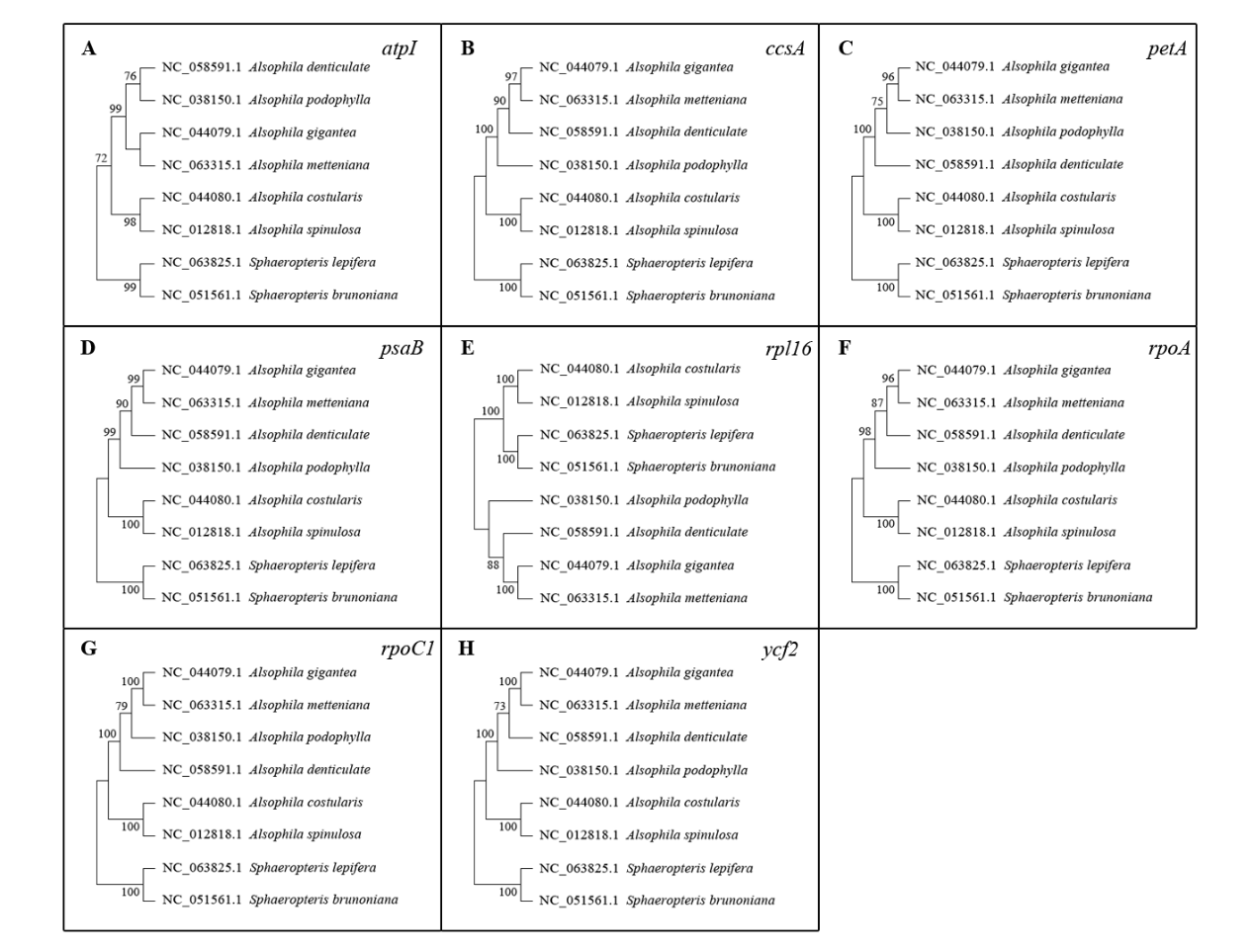


**Figure S4.** Molecular phylogenetic analysis of tree ferns.
