# Supplementary material for: Complete chloroplast genome molecular structure, comparative and phylogenetic analyses of Sphaeropteris lepifera of Cyatheaceae family: a tree fern from China
Source: Sci Rep. 2023 Jan 24;13:1356. doi: 10.1038/s41598-023-28432-3 (PMC9873718; doi:10.1038/s41598-023-28432-3)
Supplement: Supplementary file 2 — Supplementary Tables. [file 41598_2023_28432_MOESM2_ESM.docx]

**Table S1. Relative synonymous codon usage of *S. lepifera*.**

| **Amino acid** | **Codon** | **Count** | **RSCU** | **Amino acid** | **Codon** | **Count** | **RSCU** |
| --- | --- | --- | --- | --- | --- | --- | --- |
| Ala | GCU | 671 | 1.7976 | Leu | CUC | 211 | 0.5124 |
| Ala | GCA | 397 | 1.0636 | Leu | CUG | 202 | 0.4902 |
| Ala | GCC | 240 | 0.6432 | Lys | AAA | 802 | 1.3224 |
| Ala | GCG | 185 | 0.4956 | Lys | AAG | 411 | 0.6776 |
| Arg | AGA | 432 | 1.6854 | Met | AUG | 491 | 6.9860 |
| Arg | CGU | 348 | 1.3578 | Met | GUG | 1 | 0.0140 |
| Arg | CGA | 279 | 1.0884 | Phe | UUU | 696 | 1.2232 |
| Arg | AGG | 200 | 0.7800 | Phe | UUC | 442 | 0.7768 |
| Arg | CGG | 150 | 0.5850 | Pro | CCU | 353 | 1.2720 |
| Arg | CGC | 129 | 0.5034 | Pro | CCA | 318 | 1.1460 |
| Asn | AAU | 805 | 1.4730 | Pro | CCC | 281 | 1.0128 |
| Asn | AAC | 288 | 0.5270 | Pro | CCG | 158 | 0.5692 |
| Asp | GAU | 783 | 1.5660 | Ser | UCU | 533 | 1.5840 |
| Asp | GAC | 217 | 0.4340 | Ser | UCA | 409 | 1.2156 |
| Cys | UGU | 189 | 1.4052 | Ser | AGU | 389 | 1.1562 |
| Cys | UGC | 80 | 0.5948 | Ser | UCC | 315 | 0.9360 |
| Gln | CAA | 527 | 1.3814 | Ser | UCG | 232 | 0.6894 |
| Gln | CAG | 236 | 0.6186 | Ser | AGC | 141 | 0.4188 |
| Glu | GAA | 882 | 1.3792 | Ter | UAA | 38 | 1.3734 |
| Glu | GAG | 397 | 0.6208 | Ter | UGA | 27 | 0.9759 |
| Gly | GGU | 623 | 1.4808 | Ter | UAG | 18 | 0.6507 |
| Gly | GGA | 616 | 1.4640 | Thr | ACU | 489 | 1.5268 |
| Gly | GGG | 273 | 0.6488 | Thr | ACA | 360 | 1.1240 |
| Gly | GGC | 171 | 0.4064 | Thr | ACC | 269 | 0.8400 |
| His | CAU | 366 | 1.3432 | Thr | ACG | 163 | 0.5088 |
| His | CAC | 179 | 0.6568 | Trp | UGG | 397 | 1.0000 |
| Ile | AUU | 945 | 1.4796 | Tyr | UAU | 578 | 1.4184 |
| Ile | AUA | 549 | 0.8595 | Tyr | UAC | 237 | 0.5816 |
| Ile | AUC | 422 | 0.6609 | Val | GUU | 517 | 1.4420 |
| Leu | UUA | 703 | 1.7064 | Val | GUA | 516 | 1.4392 |
| Leu | UUG | 511 | 1.2402 | Val | GUG | 210 | 0.5856 |
| Leu | CUA | 429 | 1.0410 | Val | GUC | 191 | 0.5328 |
| Leu | CUU | 416 | 1.0098 |  |  |  |  |

**Table S2. Comparison of chloroplast genomes of seven tree ferns.**

| Genome | *S. lepifera* | *S. brunoniana* | *A. spinulosa* | *A. podophylla* | *A. gigantea* | *A. costularis* | *A. denticulate* |
| --- | --- | --- | --- | --- | --- | --- | --- |
| NCBI ID | NC_063825.1 | NC_051561.1 | NC_012818.1 | NC_038150.1 | NC_044079.1 | NC_044080.1 | NC_058591.1 |
| Genome size(bp) | 162,114 | 156,659 | 156,661 | 166,151 | 161,679 | 156,675 | 154,046 |
| LSC length/bp | 86,363 | 86,196 | 86,308 | 86,762 | 92,315 | 86,338 | 85,975 |
| SSC length/bp | 27,731 | 22,441 | 21,623 | 21,641 | 21,702 | 21,625 | 21,581 |
| IR length/bp | 24,028 | 24,011 | 24,365 | 28,874 | 23,831 | 24,356 | 23,245 |
| Total genes | 129 | 130 | 130 | 132 | 130 | 130 | 130 |
| Protein genes | 89 | 89 | 89 | 91 | 89 | 89 | 89 |
| tRNA | 32 | 33 | 33 | 33 | 33 | 33 | 33 |
| rRNA | 8 | 8 | 8 | 8 | 8 | 8 | 8 |

**Table S3. *Ka/Ks* analysis of chloroplast genes in seven tree ferns.**

| Gene | *A. denticulate*  NC_058591.1 | *A. gigantea*  NC_044079.1 | *A. podophylla*  NC_038150.1 | *A. spinulosa*  NC_012818.1 | *A. costularis*  NC_044080.1 | *S. brunoniana*  NC_051561.1 | Gene | *A. denticulate*  NC_058591.1 | *A. gigantea*  NC_044079.1 | *A. podophylla*  NC_038150.1 | *A. spinulosa*  NC_012818.1 | *A. costularis*  NC_044080.1 | *S. brunoniana*  NC_051561.1 |
| --- | --- | --- | --- | --- | --- | --- | --- | --- | --- | --- | --- | --- | --- |
| *accD* | 0.3456 | 0.3021 | 0.2405 | 0.2213 | 0.2213 | - | *psbC* | 0.0314 | 0.0492 | 0.0336 | 0.0350 | 0.0350 | - |
| *atpA* | 0.0892 | 0.0860 | 0.0759 | 0.0753 | 0.0753 | - | *psbD* | 0.0210 | 0.0184 | 0.0210 | 0.0286 | 0.0286 | 0.1058 |
| *atpB* | 0.0630 | 0.0941 | 0.0584 | 0.0290 | 0.0290 | - | *psbH* | 0.0387 | 0.0476 | 0.0476 | 0.0600 | 0.0600 | - |
| *atpE* | 0.0774 | 0.0326 | 0.0385 | 0.0785 | 0.0785 | - | *psbK* | 0.2645 | 0.2645 | - | - | - | - |
| *atpF* | 0.2121 | 0.2100 | 0.2629 | 0.0452 | 0.0452 | - | *psbZ* | 0.1411 | 0.1125 | 0.1956 | 0.1411 | 0.1411 | - |
| *atpI* | 0.2136 | 0.2136 | 0.2136 | 0.1260 | 0.1260 | - | *rbcL* | 0.2116 | 0.2197 | 0.2201 | 0.0879 | 0.0879 | 0.1394 |
| *ccsA* | 0.3622 | 0.3387 | 0.2911 | - | - | - | *rpl2* | 0.1151 | 0.1170 | 0.1662 | 0.0774 | 0.0968 | - |
| *cemA* | - | 0.5797 | - | - | - | 0.2801 | *rpl14* | 0.2888 | 0.2923 | 0.2938 | - | - | - |
| *chlB* | 0.1726 | 0.2184 | 0.1991 | 0.1936 | 0.1936 | 0.1388 | *rpl16* | - | - | 0.3441 | - | - | - |
| *chlL* | 0.0481 | 0.0745 | 0.0512 | 0.1325 | 0.1325 | 0.2386 | *rpl20* | 0.3631 | - | 0.3791 | - | - | - |
| *chlN* | 0.2570 | 0.2216 | 0.2564 | 0.1796 | 0.1796 | - | *rpl21* | 0.2213 | 0.2714 | 0.1809 | 0.2302 | 0.2302 | - |
| *clpP* | - | - | - | 0.0643 | 0.0643 | - | *rpl22* | 0.1142 | - | - | - | - | - |
| *infA* | 0.2110 | 0.1426 | 0.2678 | 0.1426 | 0.1426 | - | *rpl23* | 0.2109 | 0.1572 | 0.1591 | - | - | - |
| *matK* | 0.5863 | 0.5470 | 0.5352 | 0.4670 | 0.4670 | 0.2210 | *rpl33* | - | - | - | 0.1350 | 0.1350 | - |
| *ndhA* | 0.3135 | 0.3220 | 0.3458 | 0.2773 | 0.2773 | - | *rpoA* | 0.2743 | 0.2744 | 0.2309 | 0.2111 | 0.2111 | - |
| *ndhB* | 0.2040 | 0.2621 | 0.2092 | 0.2250 | 0.2250 | 0.2504 | *rpoB* | 0.1300 | 0.1279 | 0.1279 | 0.1431 | 0.1431 | 0.0586 |
| *ndhC* | - | 0.1821 | - | - | - | - | *rpoC1* | 0.2044 | 0.2305 | 0.1955 | 0.2526 | 0.2526 | - |
| *ndhD* | 0.1708 | 0.2103 | 0.1847 | 0.1317 | 0.1317 | - | *rpoC2* | 0.3671 | 0.3552 | 0.3294 | 0.3381 | 0.3381 | - |
| *ndhE* | 0.3529 | - | 0.2971 | - | - | - | *rps2* | - | - | - | 0.3596 | 0.3596 | 0.1422 |
| *ndhF* | 0.4719 | 0.5333 | 0.4626 | 0.3717 | 0.3717 | - | *rps3* | 0.2341 | 0.2472 | 0.2045 | 0.0517 | 0.0517 | - |
| *ndhG* | 0.3800 | 0.4363 |  | 0.4008 | 0.4008 | - | *rps4* | 0.1170 | 0.1623 | 0.0890 | 0.0471 | 0.0471 | - |
| *ndhH* | 0.0772 | 0.0553 | 0.0599 | 0.0376 | 0.0376 | - | *rps7-2* | 0.2604 | 0.2604 | 0.2604 | 0.2604 | 0.2604 | - |
| *ndhI* | 0.1265 | 0.2313 | 0.1328 | 0.1196 | 0.1196 | - | *rps8* | 0.0369 | 0.0312 | 0.0275 | 0.0962 | 0.0962 | - |
| *ndhJ* | 0.1766 | 0.1616 | 0.1618 | - | - | - | *rps12-2* | 0.3167 | 0.2660 | 0.3167 | 0.2156 | 0.2156 | 0.0936 |
| *ndhK* | 0.4993 | - | - | 0.2702 | 0.2702 | - | *rps15* | 0.3942 | 0.3288 | 0.2985 | 0.1865 | 0.1865 | - |
| *petA* | 0.2348 | 0.2399 | 0.3026 | 0.1110 | 0.1110 | 0.1188 | *rps16* | 0.1895 | 0.0799 | 0.1225 | - | - | - |
| *petB* | 0.0888 | 0.0970 | 0.1076 | 0.1076 | 0.1076 | - | *rps18* | 0.2035 | 0.2035 | - | - | - | - |
| *petL* | - | 0.2430 | - | - | - | - | *rps19* | 0.1956 | 0.1161 | 0.1959 | - | - | - |
| *psaA* | 0.1033 | 0.1087 | 0.1301 | 0.0746 | 0.0746 | - | *ycf1* | 0.4579 | 0.4786 | 0.4528 | 0.4679 | 0.4679 | - |
| *psaB* | 0.1477 | 0.1320 | 0.1898 | 0.1580 | 0.1580 | - | *ycf2-2* | 0.5835 | 0.4923 | 0.5498 | - | - | - |
| *psaC* | 0.1034 | 0.1034 | 0.1034 | 0.0844 | 0.0844 | - | *ycf3* | 0.2617 | 0.3740 | 0.1897 | 0.6241 | 0.6241 | - |
| *psbA-2* | 0.0574 | 0.0574 | 0.0514 | 0.1052 | 0.1052 | - | *ycf4* | 0.3397 | 0.3059 | 0.2473 | 0.2136 | 0.2136 | - |
| *psbB* | 0.0212 | 0.0234 | 0.0218 | 0.0277 | 0.0277 | 0.1094 |  |  |  |  |  |  |  |

Note: Green represents genes present in all species. Yellow represents genes only present in one species. (P < 0.05).

**Table S4. Analysis of haplotype and diversity of chloroplast genes in *S. lepifera* from 32 different geographic populations.**

| Gene region | Number of Haplotypes, H | Haplotype (gene) diversity, Hd | Nucleotide diversity, Pi | Average number of nucleotide differences, k | Tajima's D | Fu and Li's D* | Fu and Li's F* | Aberration rat |
| --- | --- | --- | --- | --- | --- | --- | --- | --- |
| *trnG - trnR* | 31 | 0.998 | 0.01539 | 10.12903 | -1.05503 | -0.73944 | -0.91634 | 8.36% |
| *atpB* | 10 | 0.706 | 0.00267 | 1.57258 | -0.90764 | -1.59451 | -1.50463 | 1.53% |
| *rps4* | 9 | 0.843 | 0.00370 | 1.88105 | 0.23921 | -0.28298 | -0.11632 | 0.98% |
| *matk* | 12 | 0.651 | 0.00090 | 1.13710 | -2.11068 | -3.37071 | -3.27911 | 0.87% |
| *psbA - trnH* | 4 | 0.286 | 0.00179 | 0.84879 | -1.20868 | -0.75852 | -0.84261 | 0.84% |
| *proB - psbZ* | 9 | 0.607 | 0.00114 | 1.49597 | -0.73908 | -0.61167 | -0.70746 | 0.61% |
| *atpA* | 5 | 0.696 | 0.00064 | 0.82661 | -0.42343 | -0.75852 | -0.78519 | 0.23% |
| *ndhF* | 2 | 0.226 | 0.00019 | 0.22581 | -0.13835 | 0.58708 | 0.41391 | 0.09% |
| *rbcL* | 2 | 0.387 | 0.00030 | 0.38710 | 0.85334 | 0.58708 | 0.70956 | 0.08% |

**Table S5. Primers for chloroplast genome of *S. lepifera*.**

| Gene | Primer name | Primer (5'-3') | Length |
| --- | --- | --- | --- |
| *psbA* - *trnH* | Forward primer | CGCGCATGGTGGATTCACAATCC | 476bp |
|  | Reverse primer | CTTATGCATGAACGTAATGCTC |  |
| *trnG* - *trnR* | Forward primer | TCTATCAAACCGATTTTCGACTAA | 661bp |
|  | Reverse primer | GCTCGGGTAGACGCTATATTGT |  |
| *atpB* | Forward primer | GCAAGGATGAGATCTGCTCG | 589bp |
|  | Reverse primer | TCCACCAATTCGGAAATTTC |  |
| *atpA* | Forward primer | CGACCCGATGAGATTAGCAG | 1299bp |
|  | Reverse primer | ACGCAACTGAACCAAGAATC |  |
| *matK* | Forward primer | TCGGATGGACCAAGCATTGA | 1268bp |
|  | Reverse primer | TCGATAAGACAGGCCGGGTT |  |
| *ndhF* | Forward primer | AAGGTCTTCGCCGCTCATGT | 1164bp |
|  | Reverse primer | GACGCAGTGAAACCAGCTGT |  |
| *rbcL* | Forward primer | GGTGTTGGATTCAAAGCTGG | 1276bp |
|  | Reverse primer | GCGGCAGCCAATTCAGGACT |  |
| *rpoB* - *psbZ* | Forward primer | AAGACGCGCAAGTAGGTTCA | 1324bp |
|  | Reverse primer | AAGTTGTGTCAGGCGACACC |  |
| *rps4* | Forward primer | CCGAGGACCTCGTTTGAAAG | 527bp |
|  | Reverse primer | CGATAGATTCCCGATTGGCA |  |
